# Characterisation of Species and Diversity of *Anopheles gambiae* Keele Colony

**DOI:** 10.1371/journal.pone.0168999

**Published:** 2016-12-29

**Authors:** Lisa C. Ranford-Cartwright, Sion McGeechan, Donald Inch, Graeme Smart, Lenka Richterová, Jonathan M. Mwangi

**Affiliations:** Institute of Infection, Immunity and Inflammation, College of Medical, Veterinary and Life Sciences, University of Glasgow, Glasgow, Scotland, United Kingdom; Fralin Life Science Institute, Virginia Tech, UNITED STATES

## Abstract

*Anopheles gambiae sensu stricto* was recently reclassified as two species, *An*. *coluzzii* and *An*. *gambiae s*.*s*., in wild-caught mosquitoes, on the basis of the molecular form, denoted M or S, of a marker on the X chromosome. The *An*. *gambiae* Keele line is an outbred laboratory colony strain that was developed around 12 years ago by crosses between mosquitoes from 4 existing *An*. *gambiae* colonies. Laboratory colonies of mosquitoes often have limited genetic diversity because of small starting populations (founder effect) and subsequent fluctuations in colony size. Here we describe the characterisation of the chromosomal form(s) present in the Keele line, and investigate the diversity present in the colony using microsatellite markers on chromosome 3. We also characterise the large 2La inversion on chromosome 2. The results indicate that only the M-form of the chromosome X marker is present in the Keele colony, which was unexpected given that 3 of the 4 parent colonies were probably S-form. Levels of diversity were relatively high, as indicated by a mean number of microsatellite alleles of 6.25 across 4 microsatellites, in at least 25 mosquitoes. Both karyotypes of the inversion on chromosome 2 (2La/2L+^a^) were found to be present at approximately equal proportions. The Keele colony has a mixed M- and S-form origin, and in common with the PEST strain, we propose continuing to denote it as an *An*. *gambiae s*.*s*. line.

## Introduction

*Anopheles gambiae sensu lato* is the major vector of malaria in sub-saharan Africa, consisting of eight morphologically indistinguishable species. *An*. *gambiae sensu stricto* exists in two molecular forms, denoted M and S, which can be distinguished by differences in a 4Mb region located centromerically on the X chromosome, including fixed SNPs within 2.3kb intergenic spacer region in the multicopy rDNA located on the X chromosome [1,2], or an M-specific insertion of a short interspersed transposable element (SINE200) [3]. Recently it was proposed that the M- and S-forms are named as separate species [4], with the M-form taking the name *Anopheles coluzzii* and the S-form retaining the name *An*. *gambiae s*.*s*.

The two molecular forms differ in their geographical distribution and ecological niches, as well as in important phenotypic traits such as resistance to insecticides and to desiccation (reviewed in Lehmann and Diabate (2008) [5]). S-form *An*. *gambiae* are distributed across most of sub-saharan Africa, usually breeding in temporary aquatic habitats, and are associated with rainy seasons. M-form *An*. *gambiae* have a similar distribution to S-form in West and Central Africa, but are apparently absent east of the Great Rift Valley; they are able to exploit permanent breeding sites such as those associated with human activity, and breed year round [5–8]. The mechanisms driving divergence and speciation of the two molecular forms do not appear to be based on post-zygotic isolation, since laboratory crosses of M- and S-forms produce fully fertile male and female offspring [4,9]. Instead, spatial segregation of the two forms in mating swarms [10–12], or assortative mating behaviour [13] probably contributes to the usually very low rates of hybridisation seen in natural *An*. *gambiae* populations where the two forms are sympatric [12,14], although hybridisation can reach up to 20% in some sympatric populations [15–17], particularly at the extremes of the geographical distribution of sympatry [18]. Genetic divergence between the two forms in nature has been extensively studied, and was found to be widely distributed across the M- and S-form genomes, supporting the separation of the two forms into species [19–23].

The Keele mosquito strain was developed approximately 12 years ago as an outbred *An*. *gambiae s*.*s*. strain for use in experimental selection of malaria-resistant and -susceptible lines [24], and was established in Glasgow in 2002 directly from Keele University, where the line was generated. The chromosomal form has not previously been investigated, and it is usually referred to as an *An*. *gambiae s*.*s*. line. Its status now that *An*. *gambiae s*.*s*. has been divided into two species is uncertain.

The line was developed by balanced interbreeding of 4 existing laboratory colonies: ZAN U, Ifakara, KIL and G3 [24]. The first three of these colonies originated in East Africa (Zanzibar in 1984; Ifakara (Tanzania) in 1996; Marangu (Tanzania) in 1975 respectively); only the G3 line is from West Africa (MacCarthy Island, The Gambia, in 1975). Therefore, 3 of the 4 strains are expected to have been S-form at their original isolation, because of their East African origin. The G3 line could originally have been M, S or even a mixture, since hybrid M/S forms have been observed in The Gambia [16,18]. The generation of the Keele line involved initial crosses of 50 individuals of each sex between the strains KIL and Ifakara, and ZANU and G3, and the offspring of these two crosses were then mated to produce the Keele strain[24]. Keele mosquitoes are therefore likely to have a mixed origin from M- and S- form parents.

Laboratory colonies of mosquitoes usually exhibit considerable loss of diversity because of small starting populations (founder effect) and subsequent fluctuations in colony size [25,26]. Although the Keele strain was originally developed as an outbred line, the level of diversity of the strain has not previously been characterised. Microsatellites, especially those on chromosome 3 where there seems to be little restriction on gene flow [27], have been used previously to examine diversity in laboratory colonies, including the G3 line [26]. These analyses revealed reduced microsatellite diversity in two laboratory colonies relative to wild-caught mosquitoes from Mali, with an eightfold reduction in mean number of alleles found in eight microsatellite loci on chromosome 3 [26]. Wild-caught mosquitoes also had an abundance of rare alleles (frequency ≤ 0.05) which are less likely to be sampled in the relatively small starting populations for laboratory colonies.

Chromosomal inversions contribute to the substructuring of *An*. *gambiae* subpopulations and their adaptation to different environments [28–31]. A much-studied large inversion polymorphism on chromosome 2L (2La or 2L+^a^) has been associated with adaptation to aridity: *An*. *coluzzii* larvae homozygous for 2La have been shown to have enhanced thermal tolerance [32], and *An*. *gambiae s*.*s*. adults have enhanced resistance to desiccation [33,34]. Allele frequencies of the 2La/2L+^a^ vary spatially and temporally with respect to the degree of humidity in East and West Africa [35]. Both the 2La and the 2L+^a^ karyotypes are found in *An*. *gambiae s*.*s*. and *An*. *coluzzii*, but with spatial variations in the frequency; the chromosomal arrangements assort independently of molecular form in the field, and probably predate the speciation process [8]. The 2La/2L+^a^ karyotypes present in the Keele line have not previously been established.

## Materials and Methods

### DNA extraction from mosquitoes

The Keele colony held at Glasgow University is usually maintained at many thousands of individuals, with an average daily pupal collection of between 200 and 500 individuals, and 2000–3000 adults per large mating cage. Mosquitoes are allowed to mate naturally within each cage. Pupae for the study were selected randomly from different pupal trays over several days. 60 pupae were collected initially over 2–3 days for analysis of colony diversity, and an additional 90 pupae were collected for the evaluation of M and S forms at a later time point.

DNA was extracted from individual pupae from the Keele line of *An*. *gambiae s*.*s*. using the DNeasy spin column protocol (Qiagen). The sex of each pupa was first determined by examination of the terminalia [36]. Pupae were frozen at -20°C overnight and then processed to extract DNA according to the manufacturer’s protocol. Each pupa generated 200μl of genomic DNA.

### Determination of M- and S-forms

Fixed single nucleotide differences in the rDNA intergenic spacer region on the X chromosome are used to define the M- and S- chromosomal forms [1,2,37]. We used a published PCR-RFLP method which amplifies a 390bp product including the polymorphic site at position 581 of the IGS rDNA region [38]; M-forms have a T in this position whereas S-forms have a C. The PCR product was then digested with HhaI (recognition site GCG^C), resulting in fragments of 257bp, 110bp and 23bp from S form, and 367bp and 23bp from M form.

3μl of DNA from each pupa was amplified in a final volume of 28μl containing 1x PCR Buffer, 1mM MgCl_2_, 0.2mM dNTPs, 12.5ng primer UN (5’-GTGTGCCCCTTCCTCGATGT-3’), 6.25ng primer GA (5’-CTGGTTTGGTCGGCACGTTT-3’), and 1 unit Taq DNA polymerase, using the reaction conditions of an initial denaturation step 94°C for 3 minutes, and then 30 cycles of 94°C for 30s, 50°C for 45s, 72°C for 60s, with a final extension step of 7 minutes at 72°C. 12μl of the PCR product was digested at 37°C overnight with 1U of HhaI enzyme in 1 x NE Buffer 4 and 1 x BSA in a 15μl reaction volume. 7μl of the digested PCR product was run on a 2% agarose gel containing ethidium bromide and visualised by UV transillumination. Undigested PCR product (5μl) was included for comparison to check for digestion; the digested product is clearly smaller than the undigested product for the M-form, and two bands are visible for the S-form (in both cases the 23bp band is not visible on a gel).

### Microsatellite analysis of chromosome 3

Four published microsatellite loci (all dinucleotide repeats) on chromosome 3 [39,40] were chosen for analysis of diversity in the Keele colony. The markers were chosen to be spread along the chromosome; their published locations are shown in Table 1. 2μl of mosquito DNA from each pupa was amplified in a final volume of 20μl containing 1x PCR Buffer, 1mM MgCl_2_, 0.2mM dNTPs, 10nM of each primer and 1 unit Taq DNA polymerase, using the reaction conditions in Table 1. 7μl of PCR product was run for 4–6 hours on high resolution gels consisting of a 4% MetaPhor agarose gel (Lonza UK), containing ethidium bromide, and visualised by UV transillumination. PCR product sizes were estimated by comparison with a 25bp ladder using band size estimation software (Labworks, UVP, UK). Sizes were pooled into bins spanning 4 bp for each locus, taking into account the repeat size of 2 bp in these microsatellites and the estimated resolution for Metaphor Agarose of 3 bp (Lonza, UK).

**Table 1:** Microsatellite markers on chromosome 3. Distance represents the cumulative genetic distances from the most distal markers, and is taken from [39].

Marker name	Distance (cM)	Forward Primer	Reverse Primer	Amplification conditions
*Ag3H93*^a^	0	TCCCCAGCTCACCCTTCAAG	GGTTGCATGTTTGGATAGCG	95°C for 5min 30 cycles of [95°C for 20s/55°C for 30s/72°C for 30s] 72°C for 10min
*Ag3H119*^b^	29.1	GGTTGATGCTGAAGAGTGGG	ATGCCAGCGGATACGATTCG	95°C for 5min 30 cycles of [95°C for 30s/53°C for 30s/72°C f°C 30s] 72°C for 10min
*Ag3H88*^b^	61.8	TGCGGCGGTAAAGCATCAAC	CCGGTAACACTGCGCCGAC	as for *Ag3H93*
*Ag3H817*^b^	93.7	ACTGGTCCGTTGCTGCGCG	ATGAGTGAATGGTGCGCTGG	95°C for 5min 30 cycles of [95°C for 30s/53°C for 30s/65°C for 30s] 65°C for 10min

^*a*^Primer sequences taken from Lanzaro *et al*., 1995.

^*b*^Primer sequences taken from Zheng *et al*., 1996

### Analysis of inversions on chromosome 2

The inversion on the left arm of chromosome 2 known as 2La / 2L+^a^ was analysed using a published PCR strategy [41]. 2μl of mosquito DNA from each pupa was amplified in a final volume of 20μl containing 1x PCR Buffer (1.5mM MgCl_2_), 0.2mM dNTPs, 10nM of each primer (Table 2) and 1 unit Taq DNA polymerase, using the reaction conditions of an initial denaturation step 94°C for 2 minutes, and then 30 cycles of 94°C for 30s, 55°C for 30s, 70°C for 45s, with a final extension step of 10 minutes at 70°C. 10μl of each PCR product was run on a 1.5% agarose gel containing ethidium bromide and visualised by UV transillumination.

**Table 2:** PCR primers used to type the chromosome 2 inversion 2La/2L+^a^ [41].

Chromosome 2 Arrangement	Forward Primer	Reverse Primer	Expected PCR product size (bp)
2La	23A2: CTCGAAGGGACAGCGAATTA	27A2: ACACATGCTCCTTGTGAACG	492
2L+a	23A2: CTCGAAGGGACAGCGAATTA	DPCross5: GGTATTTCTGGTCACTCTGTTGG	207

## Results

### Determination of M- and S-forms

150 *An*. *gambiae* Keele mosquito pupae were analysed of which 63% were female. A PCR product of 390bp for the IGS rDNA region was amplified and digested from all 150 DNA samples. All digested PCR products were 367bp, indicating that only the M-form of this locus (T at position 581) was present in the colony (M frequency: 100% (95% confidence interval 97.5–100%)). No hybrid individuals were seen.

### Microsatellite analysis of chromosome 3

The number of alleles seen at each of the 4 microsatellite markers in shown in Table 3. Some alleles were seen in only one mosquito, giving a low allele frequency of <0.05, but no allele predominated in the Keele colony for any of the 4 microsatellite loci.

**Table 3:** Allele frequencies (freq.) for least and most common alleles at each locus for the Keele colony.

Locus	n^a^	no. alleles	Freq most common allele	Freq least common allele
*Ag3H88*	25	9	0.16	0.04
*Ag3H93*	31	7	0.258	0.032
*Ag3H817*	41	4	0.463	0.073
*Ag3H119*	39	5	0.487	0.077
**mean**	**34**	**6.25**	**0.342**	**0.056**
se	3.697	1.109	0.080	0.011

^a^n = number of mosquitoes analysed

### Analysis of inversions on chromosome 2

Both the 2La and 2L+^a^ chromosomal arrangements were found to be present in the Keele colony, with similar allele frequencies (Table 4). The numbers of homozygotes and heterozygotes was not significantly different to those expected under Hardy-Weinberg equilibrium (χ^2^ = 0.446, P = 0.800).

**Table 4:** Frequency of 2La/2l+^a^ genotypes in the Keele colony (n = 161).

		Number	Proportion
Genotype	Heterozygous	84	0.522
	Homozygous 2La/2La	46	0.286
	Homozygous 2L+^a^/2L+^a^	31	0.193
Allele frequency	2La	176	0.547
	2L+^a^	146	0.453

## Discussion

Analysis of the Keele colony unexpectedly revealed only the M-form of the rDNA marker on the X chromosome used to distinguish the two forms. Three of the four laboratory strains from which the Keele strain was established originated in East Africa, and therefore were expected to be S-form, although contamination of any of the lines with the opposite rDNA form prior to the development of the Keele strain cannot be discounted. It is unclear when or why the S-form marker on the X chromosome was lost in the Keele line; the initial crosses to generate the line [24] involved progeny of two probable S-form lines mating with the offspring of a cross between a probable M and a probable S-form line: early generations of the Keele line must have had S-form individuals or hybrids. Hybrids of M- and S-forms occur readily in the laboratory, as do as back-crosses to either M- or S- form parents, and the hybrids and their backcrosses were found to be fully fertile, with similar egg batch size, hatching rate, and larval development success under laboratory conditions [9]. Observed fitness differences of M- and S-forms under laboratory conditions include minor differences in the time to hatching of eggs, with S eggs hatching slightly earlier than M [42], higher longevity of virgin female M-forms [5], and a larger body size of M-form females, which correlated in that study with larger egg batches in M-form than S-form [43]. The latter two factors could, over time, lead to increases in the frequency of M-form mosquitoes in a mixed colony. However in the face of repeated inter-form mating, it is difficult to imagine how the rDNA marker used to discriminate the M- and S-forms would remain linked to the fitness differences unless the genes responsible for these traits were strongly linked to the X-chromosome locus.

Under natural conditions in most of sub-saharan Africa, males form swarms of only one chromosomal form, and mating is generally assortative [10–13]. The mechanism for premating isolation is not fully understood, but differences in wing widths in populations where M- and S-forms mate assortatively lend support to the hypothesis that mosquitoes choose a mate based on wing-beat frequencies [44,45]. However direct measurements from M- and S-form mosquitoes failed to show significant differences in their fundamental harmonic (wing beat) frequencies [46]. Mating of *Anopheles* in the laboratory does not appear to involve typical swarm formation, and adaptation/colonisation involves selecting for mating in the restricted space of a cage (stenogamy) [47,48]. The Keele line within the Glasgow insectaries does form small swarms, and females enter the swarms to mate (unpublished observations), but the majority of mating in a colony probably occurs outside of swarms.

The Keele colony undoubtedly had a mixed M- and S-form origin, and the colony existing today is expected to have a hybrid genome with contributions from the four parent lines. It is similar in this respect to the *An*. *gambiae* PEST strain, chosen as the first *Anopheles* genome project [49]; the strain was generated in a series of crossing steps between different colonies from Kenya (S-form) and Nigeria (M-form) [https://www.vectorbase.org/organisms/anopheles-gambiae/pest]. Since this strain is commonly referred to as *An*. *gambiae s*.*s*., despite having M- and S-form heritage, we propose that the Keele strain should also continue to be referred to as *An*. *gambiae s*.*s*.; the hybrid name *An*. *coluzzii* × *An*. *gambiae s*.*s*. may be more correct, if the rules applied to the nomenclature for inter-species hybrid plants (including the F_1_, subsequent generations, back-crosses and combinations of these) were to be followed [50], but is excessively long.

Microsatellite locus analysis of the Keele colony revealed an unexpectedly high level of allelic diversity at 4 microsatellite loci, with an average of 6.25 alleles (range 4–9) seen in the 4 microsatellite loci we examined on chromosome 3. Previous analyses of laboratory colonies e.g. Norris *et al*., 2001 [26], had shown reduced diversity compared to wild-caught mosquitoes, with an average number of alleles of 2.33 (G3 colony, range 1–6) and 3.67 (Mopti colony, range 2–6), using 9 microsatellites on chromosome 3. Two of the microsatellites used in their study, *Ag3H88* and *Ag3H119*, were also used in our analysis of the Keele strain. For *Ag3H88* both the Mopti and G3 colonies (n = 32 for each) had only 2 alleles present in the published study [26], whereas in our study, 9 alleles were observed in 25 mosquitoes of the Keele line. Analysis of *Ag3H88* diversity in wild-caught mosquitoes revealed an average of 8 alleles in populations from 12 African countries (n = 967 mosquitoes), including a population (n = 23) from McCarthy Island (the origin of the G3 line) with 9 alleles at this locus [51]. For *Ag3H119*, previous characterisation of the Mopti and G3 colonies revealed 1 and 6 alleles respectively [26], compared to 5 alleles seen in the Keele colony. A previous study of wild-caught *An*. *gambiae* diversity in 9 Tanzanian locations found 10 alleles in total for this microsatellite in 638 individuals, with an average of 6.22 alleles per sample site (mosquito numbers sampled per location ranged from 30–106) [52]. Microsatellite diversity in the Keele line therefore appears to be higher than in previously-analysed laboratory colonies, although it does not reach the diversity observed in wild-caught mosquitoes, where large numbers of alleles, many at low frequencies (<0.05) are frequently observed [26,40]. This increased diversity may reflect the generation of the Keele colony by balanced interbreeding of 4 laboratory colonies, with offspring from 50 matings for each of the pairs (KIL x Ifakara and ZAN U x G3) then mated to produce the Keele line [24].

Finally the two karyotypes of the large inversion on chromosome 2 (2La/2L+^a^) are both present in the Keele colony at approximately equal frequencies, and mosquitoes appear to mate randomly with respect to this marker.
